# Deaths from Chloroform

**Published:** 1851-10

**Authors:** 


*Deaths from Chloroform.*—A lady, thirty-six years of age, of a bilio-sanguineous temperament, who had had three children, and whose health had always been satisfactory, was very much troubled with toothache. She had had four molar teeth extracted in the same sitting six or eight years previously; after which operation she had been seized with a convulsive fit. A little while ago the toothache became very distressing again, the patient had several nervous attacks, and was tormented with the idea that her dental pains exposed her to much danger.

She sought the advice of a practitioner, and consented to a new extraction, stipulating, however, that she should take chloroform. Her husband held her hand, whilst her head was leaning against her maid; but before she had inhaled any chloroform, she started up, and attempted to run away, using very incoherent language. When a little calmer, she sat down again, and a cloth, upon which a little less than two drachms of chloroform had been poured, was placed before her mouth and nose. The patient soon pointed out, by a few words, that the chloroform was beginning to take effect, and then became insensible. The operator extracted three teeth with the greatest promptitude, and only stopped when the husband directed his attention to the patient, who seemed to have fallen into an extraordinary state. On close examination, she was discovered to be quite dead, and the best directed efforts were fruitless in reviving her. (Dr. Eissen in *Gaz. Med. de Strasbourg.*)

Another case of death from the use of chloroform occurred at the Seamen's Hospital, London, on the 8th of last July, in an operation for the removal of a diseased testis.

